# Full-Body X-Ray Imaging to Facilitate Triage: A Potential Aid in High-Volume Emergency Departments

**DOI:** 10.1155/2013/437078

**Published:** 2013-09-24

**Authors:** S. P. Whiley, H. Alves, S. Grace

**Affiliations:** ^1^Department of Human Biology, University of Cape Town, Observatory, Cape Town 7925, South Africa; ^2^Lodox Systems, 7 Dartfield Road, Sandton, Johannesburg 2146, South Africa; ^3^Trauma Unit, Charlotte Maxeke Johannesburg Academic Hospital, Private Bag X 39, Johannesburg 2000, South Africa

## Abstract

The levels of traumatic injury seen in South African emergency departments (EDs) are epidemic. This is coupled with a severe lack of resources and adequately trained emergency staff. The Lodox Statscan (LS) is an X-ray scanner capable of producing rapid, low-dose, and full-body X-ray images. In this paper, a new trauma protocol—the Johannesburg trauma protocol—that implements LS scanning on entry to the ED as a triage tool is reported. A case study illustrating the use of LS to triage 63 patients in a single Saturday shift at a level 1 Trauma Centre is also presented. Because of the ability to rapidly and safely provide X-ray imaging information to support clinical decision making, the LS could be a useful tool to aid in resource allocation to improve treatment of the high levels of trauma patients that present to South African EDs daily.

## 1. Introduction

Low- and middle-income countries account for more than 90% of global deaths from injuries. Amongst these countries is South Africa which experiences a uniquely high and violent rate of trauma. Well-documented data on the exact numbers of injuries, deaths and the underlying causes are lacking. However, some figures suggest trauma loads of between 16, 357 and 24, 113 on primary (Level 1) Trauma Centres per year [1–3]. Nationally, 46% of these are attributed to homicides, 26.7% to road traffic accidents (RTA), and 9.1% to self-inflicted injury. The homicide rate alone is nine times greater than the global average [4]. 

Coupled with this significantly high rate of trauma is the severe lack of resources and staffing. South Africa's history of apartheid has left a legacy of oversubscribed, underfunded, and poorly equipped state hospitals, dealing with more than 80% of the population's health requirements with just 25% of total healthcare expenditure [5]. Funding restrictions, together with the harsh working environment, also result in a severe shortage of adequately trained emergency staff. In South Africa, the doctor to nurse ratio is twice that for Canada and almost five times that for Israel. Possibly more disturbing, South Africa has more than four times fewer doctors per 100, 000 population than countries like Canada and Australia [6].

The two effects—high patient volumes and low resource availability—often combine to create almost warfare-like trauma situations in South African emergency departments (EDs) [7]. In response, some unique trauma mechanisms have been adopted. Among these are greater reliance on a first principles diagnostic approach and a unique triage scoring system to assist with resource allocation—the South African Triage Scale (SATS) [8]. Also in use in most of South Africa's Level 1 Trauma facilities is a South African designed high-speed, full-body, and low dose X-ray machine (Lodox Statscan, Lodox System Pty Ltd, Johannesburg, South Africa—LS), which has been shown to dramatically reduce primary survey and resuscitation times [9, 10].

This report aims to examine the role of this high-speed, full-body radiography system on the trauma protocol in one of South Africa's busiest hospitals and its effect on the triage and treatment process during the frequently occurring “mini” mass disasters that characterise the South African trauma environment. This is coupled with a view to informing global protocol for isolated multiple-casualty situations and in other environments with a high trauma burden.

## 2. Methods

An examination of the trauma protocol and response in a Tertiary Level 1 Trauma hospital in Johannesburg, South Africa, was undertaken. The trauma workflow in that hospital is presented, as well as a case study illustrating the use of full-body radiography in a high-volume trauma situation. 

### 2.1. System Description

**Figure 1:**
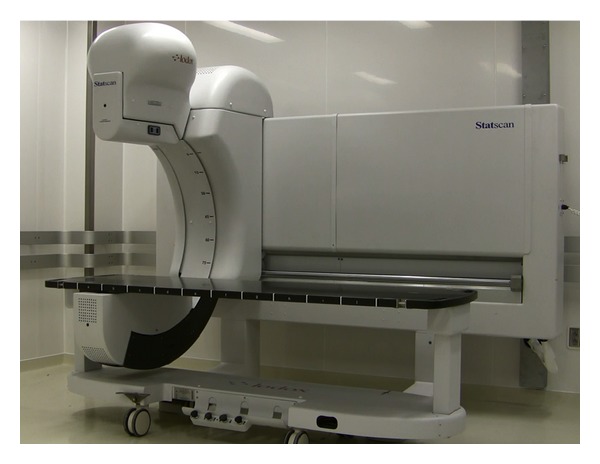
The Lodox Statscan (LS).

The LS (Figure 1) has an X-ray tube, mounted on one end of a C-arm that emits a focused, collimated fan-beam of X-rays. The X-ray detector unit, on the opposite end of the C-arm, consists of scintillator arrays optically linked to charge-coupled devices [11]. The C-arm takes 13 seconds to travel linearly along the table length, and a full-body (1.8 m) anterior-posterior digital scan is available in less than 1 minute. The C-arm can be rotated axially around the patient to any angle up to 90° to allow subsequent shoot-through lateral and oblique views to be taken. The unit includes a moveable, docking resuscitation trolley to eliminate transfer movement, which allows complete patient access for on-going resuscitation. 

Utilising linear slot scanning radiography (LSSR) and several modifications to the imaging chain, the system achieves an extremely low emitted and scattered X-ray dose [11]. The digital radiation dose relative to conventional X-ray dose varies from 72% (chest) to 2% (pelvis), with a simple average of 6% [11–13]. The radiation skin-entry dose averages 36 mrem (range 18 to 67) compared with a conventional dose of 591 mrem (range 20 to 2280) [14]. Effective doses are between 9% and 75% of the United Nations Scientific Committee Report on the Effects of Ionizing Radiation Doses for Standard Examinations [15].

## 3. Results: The Johannesburg Trauma Protocol

Previous studies have shown that this hospital admits approximately 16, 356 trauma patients each year, which is an average of more than 44 per day. 51% of the cases occur over weekends, between Friday and Sunday. Of these, those seen between 18 h00 and 08 h00 far exceed those seen during “working” hours [2]. This is consistent with studies in other parts of the country [1, 3]. Approximately 1 in 20 patients sustain multiple injuries, and 60% are classified as serious, severe, or critical [2]. 

**Figure 2:**
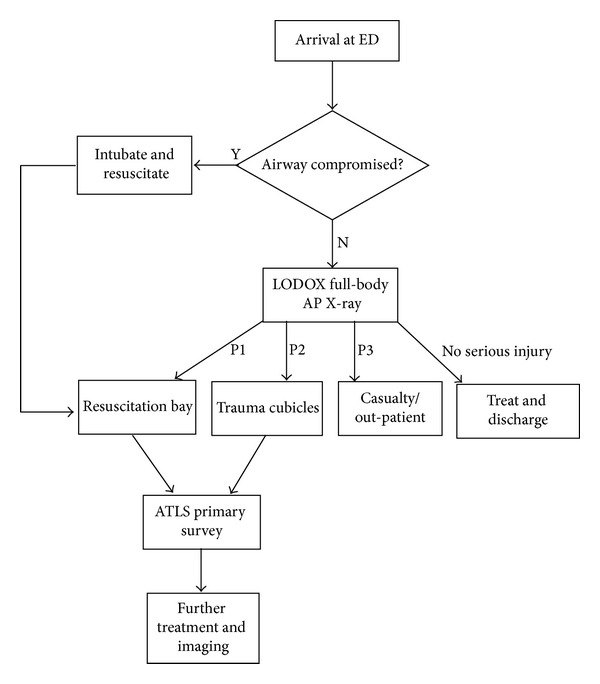
Flowchart outlining the Johannesburg trauma protocol.

The hospital has had an LS installed since 2009, which is situated directly in the resuscitation area, on the pathway between the ambulance offloading area and casualty.  Figure 2 illustrates the trauma protocol in place at CMJAH, which has been developed since the installation of this machine.

The chart shows how all casualty patients presenting to the ED, apart from those with a severely compromised airway, are scanned with the LS to obtain a full-body radiograph. ATLS and resuscitation are continued for P1 and P2 patients after this X-ray triage process. Less serious patients are discharged or rerouted to lower-priority casualty waiting areas.

## 4. Case Study: High Trauma Workload

To illustrate the use of Lodox as a triage tool, as presented in the trauma protocol of Figure 2, we present an example of the typical trauma load faced on a Saturday shift (7 am–7 pm). The staff on duty were one consultant, three doctors, a registrar, an intern, and seven nurses.

During this 24-hour period, 63 patients presented to the emergency department. On arrival, every patient received a full-body LS X-ray scan in the anteroposterior (AP) orientation. This image was used to triage patients into resuscitation (resus) bays, cubicles, and casualty/outpatient wards for treatment and discharge home. Of the 63 patients seen, 28 were classified as Priority 1 (P1) patients and taken to resus. 

Table 1 shows the numbers of patients with each kind of injury. Fractures and lacerations were the most common causes for ED arrival. It is notable that stab wounds were the third most common causes of injury with a total of 8 victims. Although the cause for each patient's injury was not always noted, it was recorded that 22 patients were the victims of motor vehicle accidents (MVAs). Whilst no “mini” mass disasters, such as minibus taxi crashes, occurred on this day, there were four occasions when 2 patients arrived simultaneously, with at least one being of P1 level. Simple averaging shows that a patient arrived at the ED every 22.8 minutes. However, the spread was not even; for instance, in the hour between 3 am and 4 am, 8 patients (4 P1) arrived at the ED.

**Figure 3:**
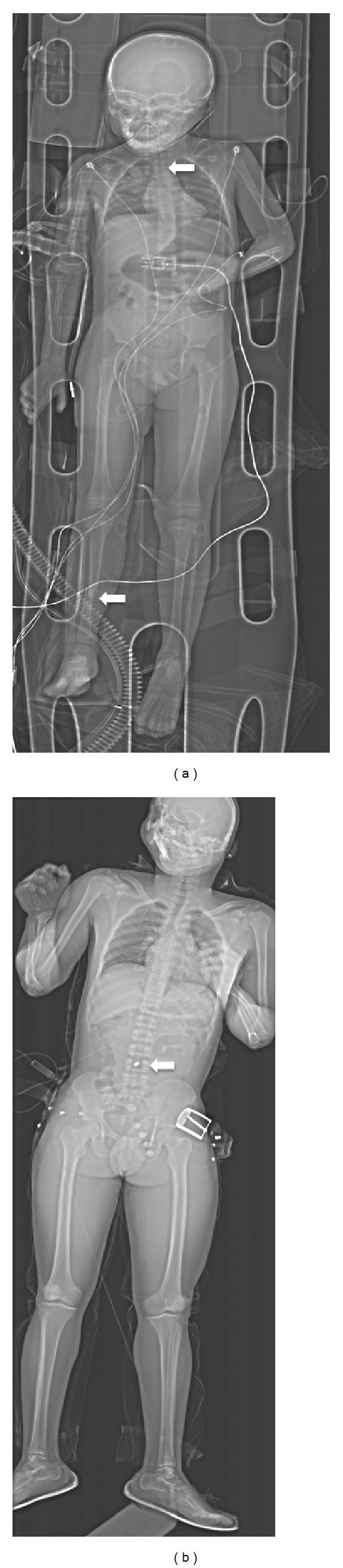
(a) LS X-ray of a paediatric patient showing fractured tibia/fibula and verification of chest intubation and (b) LS of an adult male showing gunshot wound to the abdomen.

Of the 28 P1 patients, 16 were eventually discharged home. During treatment, 18 were referred for follow-up CT imaging, 3 to orthopaedics, 1 to plastics, and 4 to other hospital departments. One patient was referred to another clinic, and 1 was not documented. All of the patients survived. Although Lodox X-ray scanning was performed within the first few minutes at the ED, the mean time between arrival and final discharge from the ED was lengthy at 12 hours and 51 minutes (minimum 1 hour and 20 minutes and maximum 72 hours). 39 patients were referred to radiology for further plain X-rays.  Figure 3 shows two full-body X-ray images of patients treated during this 24-hour shift.

## 5. Discussion

With death rates of more than 60, 000 per year and one of the highest rates of traumatic death in the world, trauma is at epidemic levels in South Africa [16]. What this means “on the ground” in emergency departments countrywide is a situation where demand exceeds capacity. In this situation, it is essential to be able to prioritise patients based on the severity of trauma to ensure that the limited resources available are used in the best possible way. In short, this means a method of accurate triaging [17]. 

Undertriage is defined as the underestimation of the severity of an illness or injury, resulting in a patient receiving lower levels of treatment than required. Historically, acceptable undertriage rates have been set at 5% or less. Conversely, overtriage is the overestimation of the severity of an illness or injury. Acceptable overtriage rates are much higher, typically up to 50% in an effort to avoid undertriage. However, the impact of a high overtriage rate is a high rate of resource mis-allocation, with the potential that a truly critical patient is compromised due to overtriage of a previous patient [18]. 

The provision of X-ray information early on in the resuscitation process has not been previously explored as a method of triage for mass disaster, “minimass disaster,” or high volume trauma scenarios. This is because X-ray imaging is often not available within resuscitation, cannot be performed on unstable or critical patients, takes a relatively lengthy time to perform (8–48 minutes [10]), and must be performed with caution due to radiation dose considerations [19]. This potentially limits its use in classifying patients early in the resuscitation process. Traditionally, X-ray information is obtained as an adjunct to the primary survey after the ABCDE process has been completed [20].

The LS system provides full-body X-rays in 13 seconds. Furthermore, it emits a very low level of scattered radiation, which means it can be situated directly within the resuscitation area without putting staff or patients at risk [10]. It therefore follows that it has the potential to be used differently to other radiation equipment within the trauma protocol. In this hospital, the X-ray information is provided as one of the first steps on arrival at the ED and, together with clinical decision-making tools, used to determine severity of injury and therefore further allocation of limited trauma resources.

Boffard et al. compared trauma-imaging times with the LS versus conventional imaging and found a reduction of 10.4 minutes (LS 29% faster) [9]. Exadaktylos et al. recorded a much higher improvement of 86.4% faster when LS was used for trauma imaging (19.2 minutes faster) [10]. These results indicate that, together with a unique physical positioning, the speed of image acquisition could prove to be useful in a situation where rapid, accurate triage is required. In this case study, imaging times were not recorded, only the overall time before discharge from the ED, which remained very lengthy when compared to previously reported numbers (an average of over 12 hours compared to 70.73 minutes) [9]. This indicates that the value of LS in this setting is as a tool to best allocate resources rather than a means to speed up trauma treatment. 

It has also been previously reported that LS imaging is much safer than conventional imaging, with doses of ionising radiation being reduced by up to 94% [11–13]. This means that the radiation exposure concerns, which might limit the use of conventional X-ray systems as a triage tool, are possibly not as applicable to LS imaging. However, most patients with orthopaedic injury were referred for further X-ray imaging after initial triage and treatment (39 patients). This indicates that conventional X-ray is still preferred for focal X-rays of injured extremities, so the LS does not replace these X-rays. In this hospital, the main reason for this is the lack of ability to print hard copy films of LS images, which the orthopaedic surgeons require before operating. Nevertheless, any exposure to ionising radiation should be viewed with caution, and the LS is no exception.

The traditional ATLS response dictates that, on arrival, the primary survey, consisting of the “ABCDE” steps of airway maintenance, breathing and ventilation, circulation, disability/neurological assessment, and exposure/environmental control, is first performed. Following that, the secondary survey, involving a head-to-toe evaluation plus history, is performed. X-ray imaging can be selected as an adjunct to the primary and/or secondary surveys, but is never usually performed before the “ABCDE” evaluation is started [20]. 

Two groups have previously reported on modified trauma imaging protocols using the LS. Pitcher et al. studied the implementation of LS at the Red Cross Children's Hospital in Cape Town, South Africa [21]. They found that the system increases efficiency in the case of paediatric polytrauma and accordingly modified their protocol from a standard CR polytrauma series (lateral cervical spine, supine chest, AP pelvis, and localised imaging of additional areas of clinical suspicion for bony injury) to an LS full-body AP X-ray, an LS lateral cervical spine, and an LS lateral view of any further areas of suspicion. They note that this time-saving protocol facilitates more comprehensive and efficient triaging, particularly in cases of mass casualty.

Evangelopoulos et al. presented their modified trauma protocol—The Bernese Modified ATLS Protocol—placing Lodox, together with ultrasound, as low-dose imaging adjuncts to the primary survey, allowing for a “*better understanding of patients' injury patterns*” [22]. They also comment that this combination of imaging, before beginning the secondary survey, may allow for a reduction in the number of CT scans required. Whilst, in this setting, reduction of CTs is suggested as a dose and cost saving to the patient (and hospital), in a developing world and/or mass casualty situation it could also be viewed as optimising resource utilisation.

The modified trauma protocol presented in this report shows the LS situated at the beginning of the ED path, before the ATLS response has begun. Its primary role is to act as a very specific triaging tool, allowing the small medical team and limited ED resources to be directed as best as possible. It does, however, also play the part of X-ray imaging adjunct to the primary survey, as in the Red Cross and Bernese Trauma Protocols. Other tools could be used to further streamline this process. The SATS also targets the triaging process as the key to streamlining trauma response. It, too, has been developed in response to the chronically high trauma rates in South Africa. Specifically for the South African setting and taking into account the staff and resource imbalances, it allows trained nursing staff to perform triage based on a four-level colour-coded system of severity [6]. The use of SATS has been shown to improve under- and overtriage rates when compared to the internationally used modified early warning score (MEWS). On average, SATS improved undertriage rates by 10.8% and overtriage rates by 4% [8]. Whilst it has been effectively applied in other developing world settings, it is unfortunate that this system is formally in place in only one province in the country, hampering its benefit to major Level 1 Trauma Centres [17]. No studies have been performed on the effect of LS imaging on under- and overtriage rates, and this would be required before definite conclusions on its role in triage can be made.

Many countries and organisations have put thought into developing response, triage, and treatment protocols for disaster preparedness since the advent of the “War on Terror” in 2001 [23]. In the developed world, this means practised responses for once-off situations of attack or disaster [23]. However, in the developing world, the number of patients being treated versus the resources is a constant and on-going challenge faced by emergency medicine practitioners. For instance, the (deservedly) much-lauded medical response to the Boston Marathon bombings “mass disaster” meant that Boston's busiest trauma centre (Brigham and Women's Hospital) was “flooded” with 31 patients in a day. This is less than half the amount reported here, on a fairly typical Saturday in a South African Level 1 trauma centre [1, 2]. This indicates the need for relevant solutions to the specific trauma problem faced in South Africa and other developing countries.

## 6. Conclusion

Effective and accurate triage is the key to dealing with the resource versus needs imbalance in the developing world. The trauma workflow and case study presented in this paper indicate the possibility that high-speed, low-dose, and full-body X-ray (Lodox/Statscan) imaging, when used on entry to the ED, could be an efficient and accurate method of triage in these underresourced situations.

## Figures and Tables

**Table 1:** The mechanisms of injury of the 63 patients presenting at the ED in a 24-hour period.

Mechanism of injury	P1 patients (number)	Non-P1 patients (number)
Fracture/s	6	10
Laceration	6	8
Stab	8	0
Soft tissue injury	0	7
Eye/orbital injury	2	4
Head injury	2	2
Degloving injury	2	0
Bite (human and dog)	0	2
Abrasion	0	2
Gunshot wound	1	0
Unrecorded	0	2

Total	28	35
